# Analysis of the evolution of competences in the clinical practice of the nursing degree*

**DOI:** 10.1590/1518-8345.2927.3231

**Published:** 2020-02-03

**Authors:** Maria Antonia Martínez-Momblán, Javier Colina-Torralva, Laura De la Cueva-Ariza, Eva Maria Guix-Comellas, Marta Romero-García, Pilar Delgado-Hito

**Affiliations:** 1 University of Barcelona, School of Nursing, L’Hospitalet de Llobregat, Barcelona, Espanha.

**Keywords:** Learning Environment, Competence, Bachelor’s Degree, Nursing Education, Nursing Students, Nursing Research, Ambiente de Aprendizagem, Competições, Escolaridade, Educação em Enfermagem, Estudantes de Enfermagem, Pesquisa em Enfermagem, Entorno de Aprendizaje, Competencias, Licenciatura, Educación en Enfermería, Estudiantes de Enfermería, Investigación en Enfermería

## Abstract

**Objective:**

to analyze the student’s progression in the acquisition of specific and transversal competences in relation to the competence dimensions.

**Method:**

the cross-sectional descriptive study was carried out in the clinical practice subjects included in the Nursing Degree. We included 323 students and we contemplated the development of competences through an ad-hoc questionnaire with 4 dimensions: delivery and care management, therapeutic communication, professional development and care management.

**Results:**

the academic results between the practice of the second and third year showed an improvement in care provision and therapeutic communication skills (Clinical Placements I: 12%-29%; Clinical Placements II: 32%-47%) and worsened in professional development and care management (Clinical Placements I: 44%-38%; Clinical Placements II: 44%-26%).

**Conclusion:**

the correlations between these two years were high in all the dimensions analyzed. The evaluation of competence progression in the context of clinical practice in nursing university studies allows us to optimize these practices to the maximum and establish professional profiles with a greater degree of adaptation to the professional future.

## Abstract (pt)

**Objetivo:**

analisar a progressão de estudantes na aquisição de competências específicas e transversais em relação às dimensões de competência.

**Método:**

este estudo transversal descritivo foi realizado no contexto das disciplinas de prática clínica do curso de enfermagem. O desenvolvimento de competências de 323 alunos foi analisado usando um questionário *ad-hoc* com quatro dimensões: provisão e gerenciamento do cuidado; comunicação terapêutica; desenvolvimento profissional; e gerenciamento do cuidado.

**Resultados:**

os resultados acadêmicos obtidos no segundo e terceiro anos apresentaram melhora nas habilidades referentes à provisão do cuidado e comunicação terapêutica (Práticas Clínicas I: 12%-29%; Práticas Clínicas II: 32%-47%) e uma piora no desenvolvimento profissional e gerenciamento do cuidado (Práticas Clínicas I: 44%-38%; Práticas Clínicas II: 44%-26%).

**Conclusão:**

as correlações entre estes dois anos foram altas em todas as dimensões analisadas. A avaliação da progressão de competências no contexto da prática clínica do curso de enfermagem nos permite otimizar estas práticas ao máximo e estabelecer perfis profissionais com maior grau de adaptação para o futuro profissional.

## Abstract (es)

**Objetivo:**

analizar la progresión del alumno en la adquisición de competencias específicas y transversales en relación con las dimensiones de competencia.

**Método:**

el estudio descriptivo transversal se realizó en los sujetos de práctica clínica incluidos en la licenciatura de Enfermería. Incluimos 323 estudiantes y contemplamos el desarrollo de competencias a través de un cuestionario *ad hoc* con 4 dimensiones: suministro y gestión del cuidado; comunicación terapéutica; desarrollo profesional; y, gestión del cuidado.

**Resultados:**

los resultados académicos entre la práctica del segundo y tercer año mostraron una mejora en la provisión del cuidado y en las habilidades de comunicación terapéutica: (Colocaciones clínicas I: 12% -29%; Colocaciones clínicas II: 32% -47%) y empeoraron en el desarrollo profesional y en la gestión del cuidado (Colocaciones clínicas I: 44%-38%; Colocaciones clínicas II: 44%-26%).

**Conclusión:**

las correlaciones entre estos dos años fueron altas en todas las dimensiones analizadas. La evaluación de la progresión de competencias, en el contexto de la práctica clínica, en los estudios universitarios de enfermería, nos permite optimizar estas prácticas al máximo y establecer perfiles profesionales con un mayor grado de adaptación al futuro profesional.

## Introduction

The Tuning Project establishes the conceptual bases for the creation of what would later be called the European Higher Education Area (EHEA) where there are deep transformations in teaching-learning processes, the role to be played by professors and students, the definition of a credit system, and the quality of academic programs, among others. The EHEA established a unifying system, especially the accreditation of nursing training, allowing the mobility of under and post-graduate students to the different universities^(^ 1 ^-^ 3 ^)^.

The EHEA incorporated an important paradigm shift from education directed to knowledge to competency-based learning. These competences tell us the degree of knowledge, know-how and know-how-to-be within a context of professional practice, a fundamental element to be able to lead the professional activity with optimal levels of quality inside and outside the Spanish State^(^ 4 ^-^ 5 ^)^. Therefore, the EHEA appears as a measure to improve the quality of the university system and must be carried out through the establishment of mechanisms and constant processes of evaluation, certification and accreditation of what is done and how it is done^(^ 6 ^)^.

The reality, both nationally and internationally, in the context of competences in the Degree in Nursing, is the implementation of different educational programs with different structures, levels, durations, certifications, professional and social recognition, and access to studies. This aspect led to the establishment of diverse and heterogeneous educational programs at both European level and in the United States (US)^(^ 3 ^-^ 5 ^)^.

A few years after the implementation of the new Royal Decree 1892/2008, the situation has changed and at present the training offers the establishment of more uniform programs in the content of academic programs^(^ 7 ^-^ 9 ^)^. 

The advantages of learning based on the student’s final competences are numerous: greater responsibility in their learning process, the use of active methodology, the design of practical material, the rationalization of resources and greater cohesion in the training curriculum. Competences represent, therefore, the axis par excellence of the teaching-learning process^(^ 10 ^-^ 12 ^)^. Following this guideline, the transformation of the studies from Diploma to Degree, during the academic year 2009/2010, forced the incorporation of a set of learning activities and assessment instruments that guarantee the formative curricula of the Degree in Nursing^(^ 13 ^)^. 

In Spain, there are documents that establish competences within the nursing profession, the one developed by the Catalan Council of Specialists and published by the Institut d’Estudis de la Salut is particularly notable; along the same lines we find the European Leonardo Da Vinci Project, led in this same country by the Santa Madrona University School of Nursing (Barcelona), which describes the framework of nursing competences in healthcare management, and also the one carried out by the Coordination and Development Unit of Nursing Research (Instituto de Salud Carlos III- Madrid), for research competencies in healthcare practice and specialized training that the health professionals from different academic levels should have^(^ 8 ^-^ 10 ^,^ 14 ^)^.

Similarly, there are many organizations that have focused internationally on analyzing the competence impact within the nursing profession. The Dedicated Education Unit (DEU), the European Federation of Nurse Educators (FINE Europe), and the European Academy of Nursing Science (EANS) stand out internationally. As regards Spain, in January 2017, the Official College of Nurses of Barcelona (COIB) promoted a meeting with representatives from different parts of the world to establish a workshop for the development of nursing competences in the excellence of care.

The current state of the issue is given extensively by the documents that establish and create the entire competence framework of the Bachelor Degree in Nursing, but there are few studies that have evaluated and analyzed the acquisition of specific and transversal competences for the professional development of nursing^(^ 10 ^-^ 12 ^)^. It is worth highlighting those studies that focus on the creation and validation of instruments used to evaluate competence acquisition, such as the Nursing Competency Scale (ECE), the Nurse Competence Scale (NCS) and the Practice Environment Scale-Nursing Work Index (PES- NWI)^(^ 15 ^-^ 18 ^)^. However, these instruments do not jointly evaluate the specific and transversal competences that currently integrate clinical practice subjects, nor the effectiveness of integrated learning activities in said subjects for the acquisition of competences (seminars, clinical skills, workshops, simulation, tutorials, etc.)^(^ 19 ^)^.

On the other hand, current studies establish various teaching methodologies in the context of clinical practice (simulation, tutorials and seminars), as evidence confirms that this methodology represents a structured set of documents prepared by the student, ordered chronologically or thematically, which demonstrates the evolution, progress and degree of compliance with the objectives set out in each delivery, reflecting at the same time the strategies of each student for inquiry, critical-reflective thinking, rigor and analysis^(^ 11 ^,^ 20 ^)^.

In the face of this situation there are three major problems: the first refers to the complexity of the competences, the second is related to the difficulty to make evaluations with the different teaching methodologies, and the third refers to the lack of tools that centralize and computerize data to be able to establish in-depth analyzes of the reliability and validity of the instruments used for their measurement^(^ 21 ^-^ 22 ^)^.

Several years after the implementation of the Bachelor in Nursing in Spain, we believe it is vital to evaluate competence acquisition within clinical practice subjects, since the existing plurality of agents involved in them and the numerous institutional cultures and professional profiles that participate make it a complex subject to guarantee that all those specific and transversal competences of the degree have been acquired^(^ 22 ^-^ 24 ^)^. This complexity can be minimized by establishing instruments that centralize information and rubric systems that unify the criteria and methodological rigor of the different dimensions that make up the learning activities^(^ 25 ^-^ 28 ^)^. 

The aim was to analyze the student’s progression as regards the acquisition of specific and transversal competences in relation to academic results, basal / average / final academic means and the dimensional correlations of the different competences in clinical practice subjects.

## Method

Descriptive, transversal, retrospective and correlational study.

The scope of study was the School of Nursing of the University of Barcelona (UB). The distribution of the curriculum in this school contemplates a total of 84 European Credit Transfer and Accumulation System (ECTS) of obligatory external practical courses. Clinical practice within the Bachelor’s Degree in Nursing is divided into four subjects, organized in such a way so as to incorporate criteria from lower to higher complexity, from the second to the fourth (and last) year. All of them seek the application and integration of specific and transversal competences in relation to nursing care in the different areas of action. We analyzed all 1800 students enrolled on the clinical practice subjects, known as Clinical Placements I (ECI) in the second year and Clinical Placements II (ECII) in the third year, of the Nursing Degree from September 2015 to June 2016, according to a report drawn up by the University in the records for said academic year. Considering the data extracted from the report, with the aim of achieving an accuracy of 5% in the estimation of a proportion through a bilateral 95% confidence interval, the dropout rate is close to 10%, assuming that the proportion of the population of nursing students taking the ECI and ECII subjects is 41%. Consecutive sampling. The final sample was 320 students with a distribution of ECI (n = 166) and ECII (n = 157) with a dropout rate of 10%, estimation accuracy of 5% and a confidence level of 95%. The calculation was made using Ene 3.0 software.

The following study variables were collected: i) Sociodemographic variables related to the center of practice: age, sex, health institution where the clinical practice is developed and the practice unit; ii) Variables related to the learning activities linked to the seminars: Reflective journal; (tool to reflect and write about the student’s learning process), Nursing process; (scientific method of the discipline to resolve the health problems that people undergo), Pharmacological management; (assessment and analysis of pharmacological treatments), nutritional assessment; (assessment of nutritional status), Techniques / procedures; (description and analysis based on the evidence of a procedure) and Ethical Dilemma (description of an ethical problem and its solution); iii) Variables related to clinical practice: which refer to the specific competences of the teaching plan: Professional Practice (PP) that includes the attitude during the performance of clinical practice, Care Delivery and Management (PGC) as a set, and the management of activities and services performed during the period of clinical practices, Therapeutic Communication (TC) as the ability to exchange or transmit thoughts, feelings and ideas between student-team and user, Professional Development (DP) that achieves growth and self-realization, and Care Management (CG) that includes the ability to establish scientific systematization in what is done and how it is done.

Two instruments were used to collect the variables:

- Ad-hoc form for the collection of sociodemographic variables and the sample practice center.
- ECI and ECII clinical practice questionnaires. The questionnaires for ECI contain 23 items, and there are 25 for ECII. Each of the items has response options based on a Likert-type scale with 10 response options ranging from 1 (does not perform it or performs poorly) to 10 (it is excellent, or perfect), with a maximum score of 230 and 250, respectively. Each questionnaire has 4 to 5 dimensions related to care.

In the context of clinical practice, a total of two evaluations were made for ECI (ECI0, ECI1) and three for ECII (ECI0, ECI1 and ECI2), in both the score is collected by means of a Likert scale from 1 to 10.

For the description of all the quantitative variables, the mean and the standard deviation (SD) were calculated, or the median and the interquartile range (IQR) as a function of the distribution of the data, and for the qualitative variables the frequencies and percentages were expressed. To analyze the relationship between the competence level, sociodemographic data, practice center, academic results and the teaching methodology of ECI-II in its different dimensions, an inferential analysis was carried out, using a 95% confidence level. The goodness-of-fit test was applied to check the normal distribution and parametric or non-parametric tests were used. If they did not follow a normal distribution, the variables were compared using the Chi-square, Kruskal-Wallis or Mann-Whitney test and the Spearman correlation coefficient. If they followed a normal distribution, they were compared using the Chi-square test, Student’s t test and Pearson’s correlation coefficient. The statistical program IBM SPSS Statistics 21 was used.

The recommendations of Organic Law 15/1999, of December 13 (BOE Num. 298, of December 14, 1999), on Personal Data Protection, were taken into account. An information document about the project and its objectives was provided and informed consent was obtained from students and teachers. Permission was granted by the Directorate of the School of Nursing and the approval of the Ethics Committee of the UB.

## Results

The ECI students *(n = 166)* had a mean age of 22.60 years *(SD = 5.40),* of which 80% were women *(n = 134)* and the average of those in ECII *(n = 157)* was 23.9 years *(SD = 5.02),* of which 82% were women *(n = 129).* The students performed the internships in 11 centers of the Health Institution Network of the Catalan Health Institute and predominantly in internal medicine units 15% *(n = 23),* surgeries *(n = 53)* and others *(24%).*

**Figure 1:**
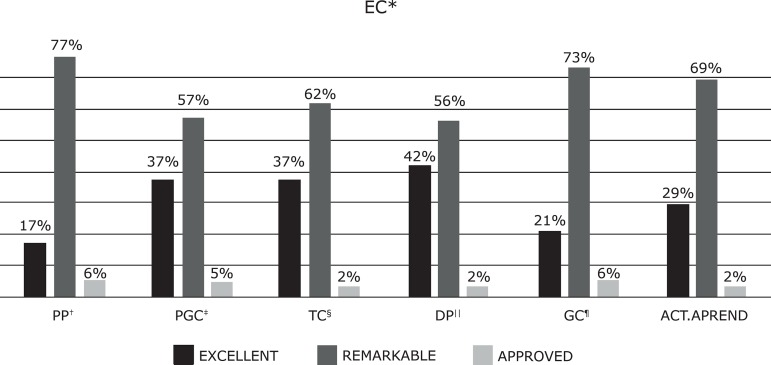
Final Academic Results for ECI* *ECI = Clinical Placements I; ^†^PP = Professional Practice; ^‡^PGC = Care Delivery and Management; ^§^TC = Therapeutic Communication; ^II^DP = Professional Development; ^¶^GC = Care Management

**Figure 2:**
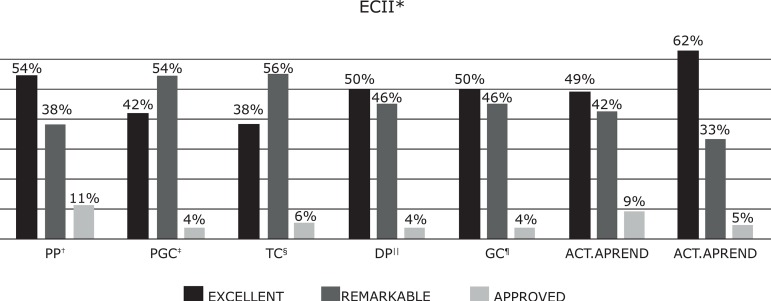
Final Academic Results for ECII* *ECII = Clinical Placements II; ^†^PP = Professional Practice; ^‡^PGC = Care Delivery and Management; ^§^TC = Therapeutic Communication; ^II^DP = Professional Development; ^¶^GC = Care Management

The final grades of the subjects for ECI and ECII did not show statistically significant differences in terms of the students’ academic results in the different evaluated dimensions *(p = <0.109),* where 90% obtained an average grade between excellent and excellent, with a very balanced distribution by dimensions (Figures 1-2). The percentage of failures in ECI and ECII was similar *(*ECI 1.36%, ECII 1.25%).

**Figure 3:**
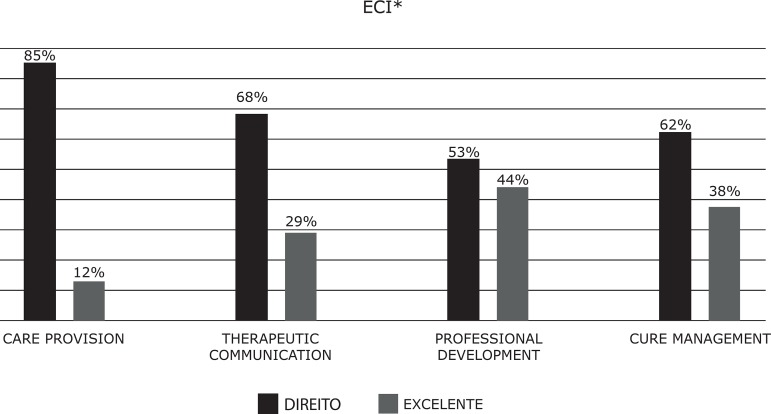
Academic results according to competence dimensions for ECI* *ECI = Clinical Placements I

**Figure 4:**
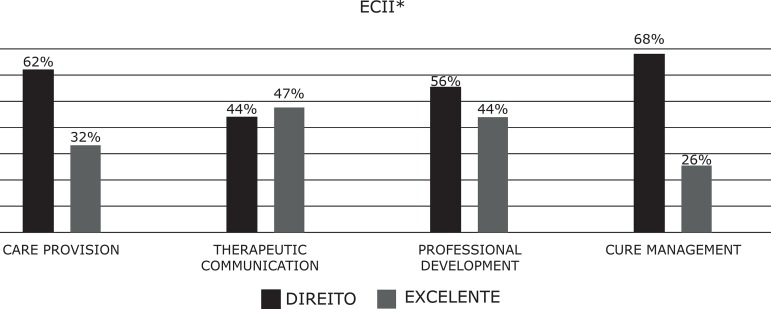
Academic results according to competence dimensions for ECII* *ECII = Clinical Placements II

The academic results between ECI and ECII; that is, between the second and third years, improved for care delivery and therapeutic communication (ECI: 12% -29%, ECII: 32% -47%), and worsened in professional development and care management (ECI: 44% -38%; ECII: 44% -26%) (Figures 3-4). 

The mean evaluation between ECI0 and ECI1, from the completion of the ECI Ad-hoc Questionnaire, showed a progression of 1.3; for the completion of the Ad-hoc Questionnaire ECII, the progression between ECI0 and ECI1 progressions was 0.8 and between ECI1 and ECI2 it was 0.52. There is a very similar improvement in each of the dimensions and at each point of determination ECII0, ECII1 and ECII2.

The dimensions analyzed, from the completion of the Ad-hoc Questionnaire in ECI1 and ECII2, showed a high correlation coefficient in the dimensions linked to the practice (ECI1: PGC *(r = 0.77),* TC *(r = 0.77 ),* DP *(r = 0.80)* and GC *(r = 0.58),* ECII2*: (r = 0.91),* TC *(r = 0.90),* DP *(r = 0.84)* and GC *( r = 0.84);* however, a low correlation coefficient was obtained in the dimensions related to the complementary learning activities of EC I1 and ECII2 (EC I1 seminars *(r = 0.56),* nursing process *(r = 0.33),* ECII2: seminars *(r = 0.51),* and disease process assessment *(r = 0.36).*

The correlations between ECI and ECII were high in all the dimensions analyzed, obtaining for professional practice *r = 0.89;* delivery and care manageme*nt r = 0.84;* therapeutic communication *r = 0.88* and professional development *r = 0.90.*

## Discussion

The findings obtained in respect to the academic results reinforce the evidence consulted, where academic qualifications maintain higher means in the results linked to the clinical practice of both ECI and ECII^(^ 29 ^-^ 30 ^)^ *.* This aspect, according to some authors, may be due to the fact that the different agents in charge of evaluating the student find it difficult to penalize low performance in the context of the practice^(^ 15 ^-^ 16 ^,^ 29 ^)^ *.*

Other authors consulted establish that the impact of the first contact in the institutional context for the student requires a higher level of adaptation and acceptance, generating a filter that triggers a greater number of failures and dropouts at the beginning of their clinical practice. In our study, the results are similar, and we believe that the reason is the progression and consequently the increase of competence demands between second and third year in the context of the different practice units. Even so, we detected that failure rates are low (ECI 1.36%, ECII 1.25%), meaning that students progressed and passed the different clinical practice subjects, despite their low performance, as confirmed by different authors consulted^(^ 30 ^-^ 33 ^)^. 

The evidence consulted confirms that students improve in therapeutic communication during the development of clinical practice, and we have not found studies that provide information on a student’s evolution in the professional development dimension and care management^(^ 26 ^,^ 34 ^)^. 

The academic results revealed high final means for all the dimensions analyzed with high progression among the different evaluations made throughout the clinical practice (ECI0, ECI1, ECI0, ECII1 and ECII2), this aspect confirms the need to establish transversal cuts in student evaluations when internship periods are very long. This monitoring makes it possible to monitor more closely, it raises the student’s awareness of those aspects that must be improved until the end of the practice and enables the associate tutor of the practice in the learning acquisition process to personalize the adaptation^(^ 31 ^-^ 32 ^)^. 

The instruments used for competence assessment in the context of clinical practice, confirm what different authors state when they refer to the need to use different evaluation methods to evaluate Miller’s pyramid, 17 through different learning activities^(^ 11 ^,^ 19 ^-^ 20 ^)^.

The different dimensions analyzed for competency acquisition were evaluated with different evaluation instruments, detecting that those dimensions measured in clinical practice had a high uniform correlation, compared to those dimensions measured in the seminar through other learning activities such as cases, reflective diary, observation activities, etc. This aspect becomes complex when the evaluations of the learning activities must be evaluated by different agents (reference nurse and associate tutor of clinical practice) giving well-differentiated academic results, making it necessary to reflect on the need to unify the reflective gaze in the practice^(^ 23 ^-^ 25 ^,^ 33 ^)^.

According to literature, the importance of the clinical facilitator in the context of clinical practice, as an agent that promotes ‘reflection in action’, is fundamental to establish cooperative learning and a collaborative approach that fosters integration between the theory and practice of the different agents involved, mainly students^(^ 26 ^-^ 28 ^)^. 

There are many authors who establish the importance of combining evaluation instruments to unify the triangulation of knowledge, skills and attitudes and thus be able to establish a teaching plan that contemplates not only all the domains of learning, but above all, be able to adapt them to the progression the student makes throughout their degree studies in said triangulation process in their academic training^(^ 13 ^)^. 

## Conclusion

The academic results of students in the context of clinical practice maintain high academic means, with a low failure rate in both ECI and ECII.

The instruments used are adequate for competency acquisition in the different dimensions analyzed, both in ECI and in ECII, detecting a different view in the marks given by the nurse compared with those granted by the associate.

Learning activities in the context of practice and seminars should be unified by the methodologies used in both contexts to reduce the low correlation between the institutional tutor or reference nurse and the academic or associate tutor in both subjects. The correlations between the competence dimensions of ECI and ECII are high, an aspect that facilitates the analysis and evaluation of the progression of these dimensions between the second and third years.

The study allowed us to carry out the joint analysis of all the competence dimensions that intervene in clinical practice subjects in the Nursing Degree. This analysis serves to establish future lines of research that allow the validation and reliability of the assessment instrument used to measure competence dimensions.
